# Interlayer Modification of Crystalline Layered Silicates with Oligodimethylsiloxane

**DOI:** 10.1002/chem.202500262

**Published:** 2025-04-13

**Authors:** Riho Wakino, Mai Suzuki, Yoshiaki Miyamoto, Masashi Yatomi, Takamichi Matsuno, Atsushi Shimojima

**Affiliations:** ^1^ Department of Applied Chemistry Faculty of Science and Engineering Waseda University Tokyo Japan; ^2^ Kagami Memorial Research Institute for Materials Science and Technology Waseda University Tokyo Japan; ^3^ Waseda Research Institute for Science and Engineering Waseda University Tokyo Japan

**Keywords:** layered silicate, nanobuilding blocks, nanosheets, self‐healing, silylation

## Abstract

Crystalline layered silicates are valuable precursors for the synthesis of functional siloxane‐based nanomaterials. In this study, vinyl‐terminated oligodimethylsiloxane was grafted onto the interlayer surfaces of a crystalline layered silicate, octosilicate. The oligosiloxane modification facilitated the delamination of the layers in hydrophobic organic solvents. Hydrosilylation reactions between the oligosiloxane‐modified nanosheets and SiH‐terminated polydimethylsiloxane resulted in a clear, stretchable elastomer, demonstrating that the nanosheets acted as cross‐linkers. Furthermore, the introduction of silanolate groups into the elastomer imparted self‐healing properties. These findings expand the potential of crystalline layered silicates as nanobuilding blocks for new materials.

## Graphical abstract

Vinyl‐terminated oligodimethylsiloxane chains are grafted on the interlayer surfaces of a crystalline layered silicate. Delaminated nanosheets are used as novel siloxane‐based nanobuilding blocks to produce nanocomposite silicone elastomers.

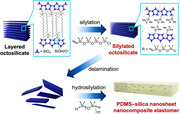

## 1 Introduction

Siloxane‐based materials are widely used in various applications owing to their excellent heat and weather resistance. A broad range of material properties can be achieved by controlling the siloxane (Si─O─Si) structures, and using siloxane compounds with well‐defined structures as building blocks provides a rational approach. Oligosiloxanes, such as cage‐type siloxanes^[^ ^1^, ^2^ ^]^ and cyclic siloxanes,^[^ ^3^, ^4^ ^]^ are commonly used as molecular building blocks to produce nanoporous materials and inorganic–organic hybrid materials. By utilizing larger siloxane compounds with higher‐order structures, unique properties, and functions based on their architectures can be realized.

Crystalline layered silicates composed of two‐dimensional (2D) nanosheets of SiO_4_ tetrahedra, with exchangeable cations between the layers, are attractive as a precursor of 2D nanobuilding blocks. The presence of reactive SiOH/SiO^−^ groups regularly arranged on the interlayer surfaces distinguishes them from other 2D inorganic compounds, such as clay minerals. The structures and compositions of the silicate layers can be varied by silylation of the interlayer SiOH/SiO^−^ groups using various organo(chloro/alkoxy)silanes and alkoxychlorosilanes.^[^ ^5^, ^6^, ^7^, ^8^ ^]^ Microporous three‐dimensional (3D) frameworks have been constructed through interlayer linking by dehydration condensation of the silanol groups between adjacent layers^[^ ^9^, ^10^, ^11^, ^12^, ^13^ ^]^ or by silylation with organo‐bridged silanes.^[^ ^14^, ^15^ ^]^ Na‐type layered octosilicate (Na_8_Si_32_O_64_(OH)_8_·32H_2_O)^[^ ^16^, ^17^ ^]^ has been widely utilized because of the relatively rigid framework (consisting of four‐, five‐, and six‐membered siloxane rings) with a well‐established crystal structure.

Delamination of layered silicates allows their use as 2D nanobuilding blocks with high aspect ratios, high surface area, and high dispersibility. Functional coatings and films can be obtained by stacking the dispersed nanosheets. Loch et al. recently achieved the monolayer exfoliation of layered octosilicate in water through osmotic swelling via ion exchange of the interlayer Na^+^ ions with meglumine cations.^[^ ^18^ ^]^ The exfoliated nanosheets were stacked onto a substrate to create uniform silica coatings with high gas barrier properties. Awaya et al. reported the preparation of monolayer nanosheets by ultrasonication of H‐type octosilicate in a methanol aqueous solution containing tetrabutylammonium ions, showing that the resulting self‐standing films function as proton conductive membranes.^[^ ^19^ ^]^

Surface silylation of silicate nanosheets is crucial for diverse functionalization. Direct silylation of delaminated nanosheets dispersed in water is challenging because of the deterioration of the silylating agents via hydrolysis. A few reports demonstrated the delamination of layered silicates after organosilylation. Takahashi et al. reported that layered octosilicate modified with butylimidazolium groups was fully delaminated in water.^[^ ^20^ ^]^ Nomi et al. reported the delamination of layered octosilicate modified with silyl groups containing phosphonic acid groups, which act as solid acids.^[^ ^21^ ^]^ In both cases, monolayer exfoliation was achieved via ultrasonication, leading to the fragmentation of the nanosheets. Furthermore, the modified silyl groups were specifically designed for dispersion in protic solvents such as water and methanol, which limits their use as nanobuilding blocks.

**Scheme 1:**
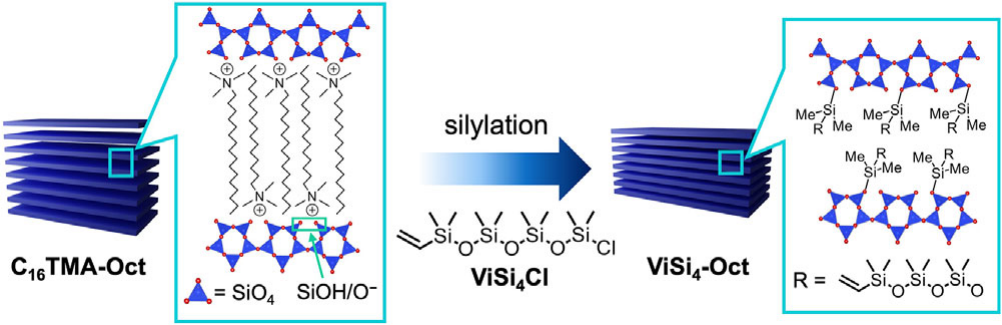
Interlayer modification of layered octosilicate with vinyl‐terminated oligodimethylsiloxane.

Herein, we report the interlayer modification of layered octosilicate by silylation with oligodimethylsiloxane containing terminal vinyl and SiCl groups (**ViSi_4_Cl**) (Scheme 1). The interlayer‐expanded octosilicate, prepared by ion exchange of Na^+^ with hexadecyltrimethylammonium cations (**C_16_TMA‐Oct**),^[^ ^6^, ^7^, ^8^ ^]^ was used as an intermediate. The oligodimethylsiloxane‐modified octosilicate (**ViSi_4_‐Oct**) underwent delamination by simple stirring in nonpolar organic solvents. Furthermore, to demonstrate the use of the delaminated layers as a nanobuilding block, a siloxane‐based nanocomposite elastomer (**Oct‐PDMS**) was prepared through the hydrosilylation reaction with SiH‐terminated polydimethylsiloxane (PDMS). PDMS‐based elastomers have many applications owing to their flexibility, biocompatibility, and electrical insulation.^[^ ^22^ ^]^ Nanocomposites of inorganic nanosheets and polymers, including PDMS, typically exhibit enhanced thermal, mechanical, and barrier properties.^[^ ^23^, ^24^ ^]^ In contrast to previously reported PDMS–clay nanocomposites that rely on non‐covalent surface interactions,^[^ ^24^, ^25^ ^]^ the silicate nanosheets in **Oct‐PDMS** serve as both 2D inorganic fillers and cross‐linkers, forming a novel covalently cross‐linked siloxane‐based material. In addition, we also examined the introduction of silanolate (SiO^−^) groups to confer self‐healing properties.

## 2 Results and Discussion

**Figure 1:**
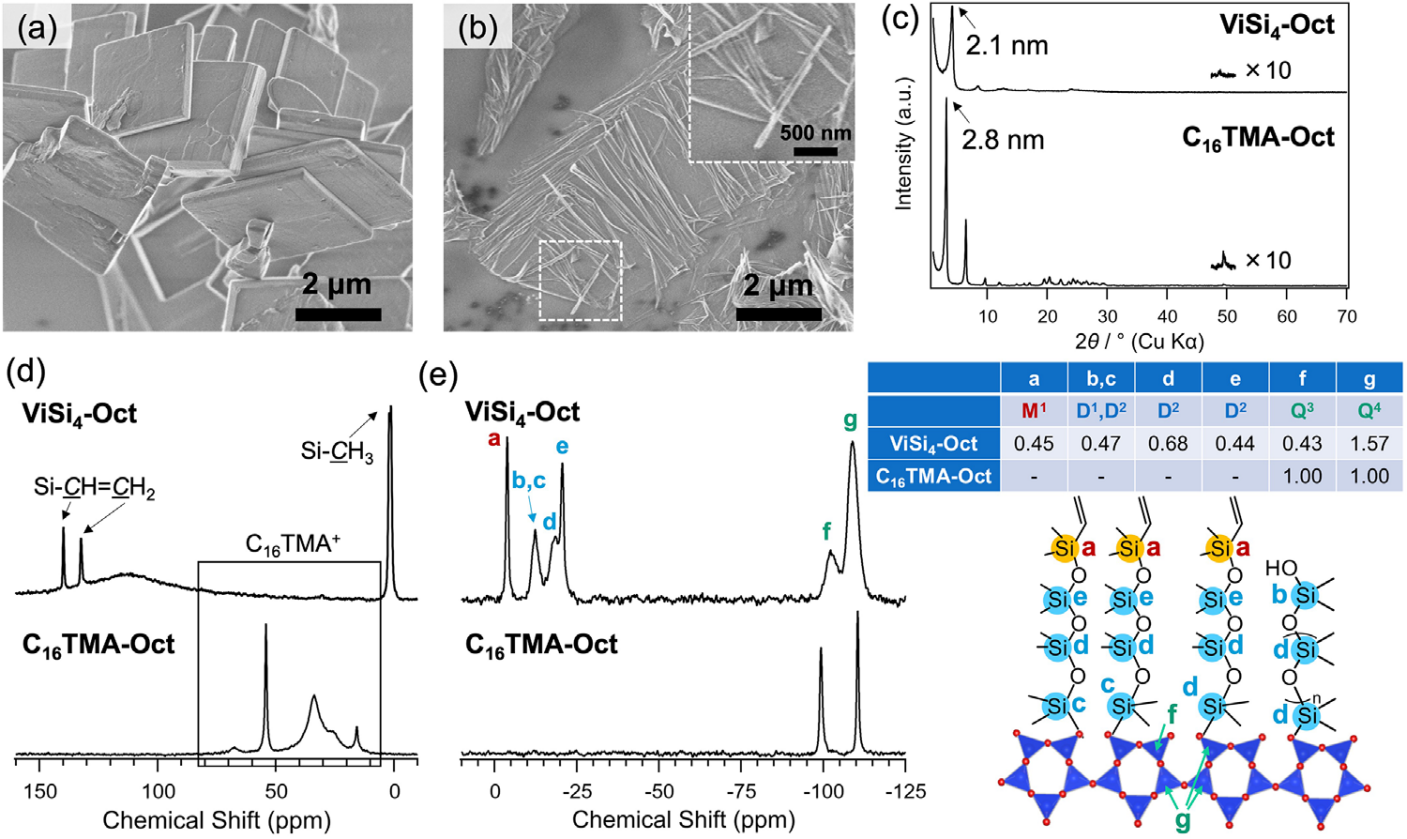
SEM images of (a) **C_16_TMA‐Oct** and (b) **ViSi_4_‐Oct** (inset: enlarged image), (c) powder XRD patterns of **C**_**16**_ **TMA‐Oct** (bottom) and **ViSi**_**4**_ **‐Oct** (top), (d) ^13^C cross‐polarization (CP)/MAS NMR spectrum of **C_16_TMA‐Oct** (bottom) and ^13^C MAS NMR spectrum of **ViSi_4_‐Oct** (top), and (e) ^29^Si MAS NMR spectra of **C_16_TMA‐Oct** (bottom) and **ViSi_4_‐Oct** (top). The insets in (e) show the integral intensity ratios of the observed signals and their assignments. The oligodimethylsiloxane chains are depicted in an extended state for clarity.

Scanning electron microscopy (SEM) observation showed that the crystal morphology of layered octosilicate was altered by silylation with **ViSi_4_Cl**. **C_16_TMA‐Oct** (Figure 1a) exhibited a square, plate‐like morphology characteristic of layered octosilicate, whereas **ViSi_4_‐Oct** (Figure 1b and Figure S1) primarily consisted of curved, plate‐like particles with misaligned layers, as well as some scrolled layers (described later) on the surface.

The powder X‐ray diffraction (XRD) patterns (Figure 1c) showed that the basal spacing of **C_16_TMA‐Oct** (*d* = 2.8 nm, which corresponds to an interdigitated monolayer arrangement of interlayer C_16_TMA cations) decreased to 2.1 nm for **ViSi_4_‐Oct**. The small peak at 2*θ* = 49°, corresponding to the atomic scale periodicity of the silicate layers in the in‐plane direction, was still observed after silylation, suggesting that the crystallinity of layered octosilicate was at least partially retained.

The solid‐state ^13^C magic‐angle spinning (MAS) nuclear magnetic resonance (NMR) analysis (Figure 1d) confirmed that the signals corresponding to the C_16_TMA cations in **C_16_TMA‐Oct** almost disappeared, and new signals assignable to the Si*C*H_3_ groups (1.2 ppm) and the vinyl group (132.4 and 139.9 ppm) originating from **ViSi_4_Cl** were observed for **ViSi_4_‐Oct**. These results suggested that silylation occurred with the removal of C_16_TMA cations from the interlayers.

Detailed information about interlayer silylation was obtained by solid‐state ^29^Si MAS NMR analysis (Figure 1e). **C_16_TMA‐Oct** consists of Q^3^ and Q^4^ units (Q*^x^*: *Si*(OSi)*_x_* (OH/O^−^)_4−_ *_x_*, *x* = 3, 4) with a 1:1 ratio. After silylation (**ViSi_4_‐Oct**), the integral ratio of Q^3^/(Q^3^ + Q^4^) decreased, and signals corresponding to the M^1^ unit (Vi*Si*Me_2_OSi) and D^2^ units (*Si*Me_2_(OSi)_2_), which were assignable to the grafted vinyl‐terminated dimethylsiloxane chains, appeared. Based on the Q^3^:Q^4^ ratio of 0.43:1.57, the degree of silylation was calculated to be 57%. On the surface of the silicate layers of octosilicate, pairs of confronting SiOH/SiO^−^ groups (Q^3^ sites) are regularly arranged (Scheme 1, left). When one of the paired SiOH/SiO^−^ groups is silylated, the degree of silylation becomes 50%. The silylation degree of 57% indicates that at least 14% of the paired SiOH/SiO^−^ groups are both silylated.

The ^29^Si NMR signal around −12 ppm can be attributed to the O*Si*Me_2_OH groups (D^1^ units) formed by partial cleavage of the Si─O─Si bonds in **ViSi_4_Cl** and/or to the OSiMe_2_(OSi) groups (D^2^ units) bonded to the confronting SiOH/SiO^−^ sites (inset of Figure 1e). The former was consistent with the fact that the ratio of the M^1^ unit to the total D units in **ViSi_4_‐Oct** (0.28) was lower than the ratio of the ViSiMe_2_O group to the OSiMe_2_(O/Cl) groups in**ViSi_4_Cl** (0.33). The cleavage possibly occurred by the attack of nucleophiles such as pyridine and the surface SiOH/SiO^−^ groups during silylation. As for the latter possibility, the two D^2^ units bonded to the confronting SiOH/SiO^−^ sites might be observed at a lower magnetic field than typical D^2^ units due to the strained Si─O─Si bonds, which result from the steric repulsion between the adjacent oligosiloxane chains. A similar shift was also observed for the M^1^ signal (Me_3_ *Si*OSi) in the ^29^Si MAS NMR spectrum of layered octosilicate silylated with chlorotrimethylsilane.^[^ ^26^ ^]^

**Figure 2:**
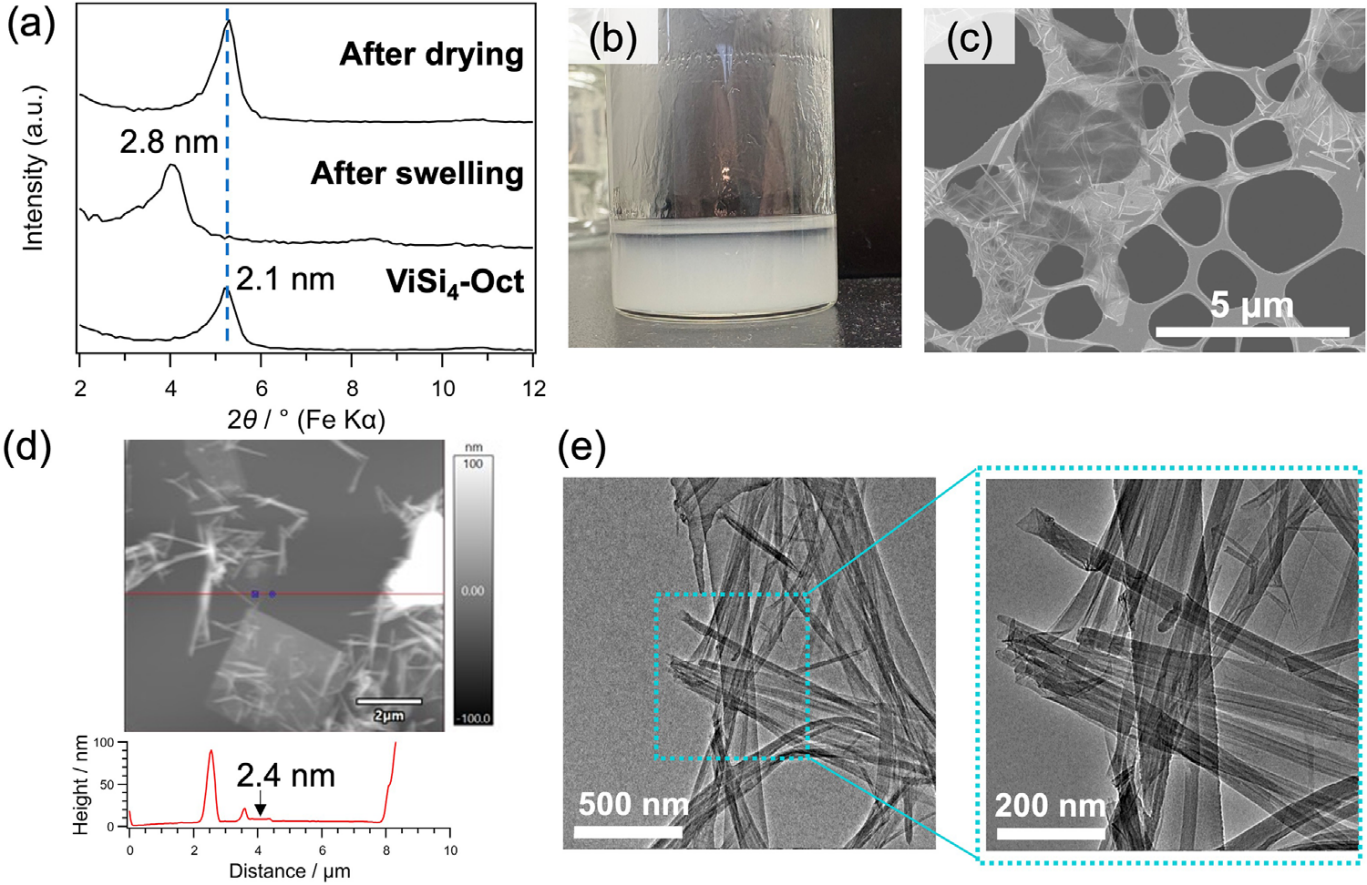
(a) XRD patterns of **ViSi_4_‐Oct** before and after swelling with cyclohexane. (b) Appearance of **ViSi_4_‐Oct_NS** (after standing for 1 day), (c) SEM image of **ViSi_4_‐Oct_NS**, (d) AFM image and the corresponding height profile of a delaminated nanosheet in **ViSi_4_‐Oct_NS**. (e) TEM images of rod‐like particles in **ViSi_4_‐Oct_NS**.

To investigate the swelling behavior of **ViSi_4_‐Oct**, cyclohexane was dropped onto the powder, and the change in basal spacing was examined. The XRD patterns (Figure 2a) revealed that the basal spacing of **ViSi_4_‐Oct** increased from 2.1 to 2.8 nm upon the addition of cyclohexane. After drying, the basal spacing returned to the original value. These results clearly demonstrate the swelling of the layers by the intercalation of cyclohexane molecules.

A translucent suspension (**ViSi_4_‐Oct_NS**) was obtained by stirring the powders of **ViSi_4_‐Oct** in cyclohexane at room temperature for 2 days. Only slight sedimentation was observed even after standing the suspension for 1 day (Figure 2b). The SEM observation (Figure 2c) showed the presence of nanosheets that are much thinner than the plate‐like particles observed for **ViSi_4_‐Oct** (Figure 1b). Some rod‐like particles were also observed. The atomic force microscopy (AFM) observation (Figure 2d and Supporting Information Figure S2) showed that the thickness of most of the nanosheets (excluding the rod‐like particles and aggregated particles) was less than 10 nm, with a small number of nanosheets having a thickness of 20–30 nm. The average thickness, calculated from the measurement of 48 nanosheets, was ∼4.2 nm. The thinnest layer was ∼2.3 nm in thickness, which roughly corresponds to the thickness of a single layer of **ViSi_4_‐Oct** (*d* = 2.1 nm).

The transmission electron microscopy (TEM) image of the rod‐like particles (Figure 2e) showed a strong contrast on the outer part and a faint contrast in the center, suggesting the formation of a tube‐like structure. It was considered that the rod‐like structures were formed through the rolling up of delaminated nanosheets into nanoscrolls. The scrolling might occur during the drying of **ViSi_4_‐Oct_NS** for electron microscopy observations, though the details remain unclear.

Swelling and delamination of **ViSi_4_‐Oct** were observed in other solvents such as *n*‐hexane and toluene. When *n*‐hexane and toluene were dropped onto the powder of **ViSi_4_‐Oct**, the basal spacing increased from 2.1 to 2.8 and 2.7 nm, respectively (Supporting Information, Figure S3a). After stirring **ViSi_4_‐Oct** in these solvents for 3 days, translucent suspensions were obtained, and sedimentation after 1 day of standing was more pronounced in toluene (Figure S3b,d). The SEM images (Figure S3c,e) showed that thin nanosheets with some scrolls were formed in *n*‐hexane, similar to those observed for **ViSi_4_‐Oct_NS**, while relatively thick plates were also present in toluene. These results suggested that the degree of delamination was greater in non‐polar *n*‐hexane and cyclohexane than in toluene.

To verify the role of the grafted vinyl‐terminated oligodimethylsiloxane chains in swelling and delamination of the layers, dimethylvinylchlorosilane (ViMe_2_SiCl) and dimethyl‐*n*‐octylchlorosilane (C_8_Me_2_SiCl) were used for interlayer silylation of **C_16_TMA‐Oct** (Supporting Information, Procedure S1 and Figures S4–S6). ViMe_2_Si‐modified octosilicate (**ViMe_2_Si‐Oct**) showed no swelling with cyclohexane, which can be attributed to the smaller *d‐*spacing (*d* = 1.5 nm, Figure S5a) and/or the absence of dimethylsiloxane chains. C_8_Me_2_Si‐modified octosilicate (**C_8_Me_2_Si‐Oct**), which had a similar *d*‐spacing to **ViSi_4_‐Oct** (*d* = 2.1 nm, Figure S5a), underwent swelling and partial delamination in cyclohexane. However, the thickness was typically on the order of tens of nanometers (Figure S6). These results suggested that oligodimethylsiloxane chains play a significant role in the delamination of **ViSi_4_‐Oct**, probably due to their high mobility and relatively weak intermolecular interactions.

**Figure 3:**
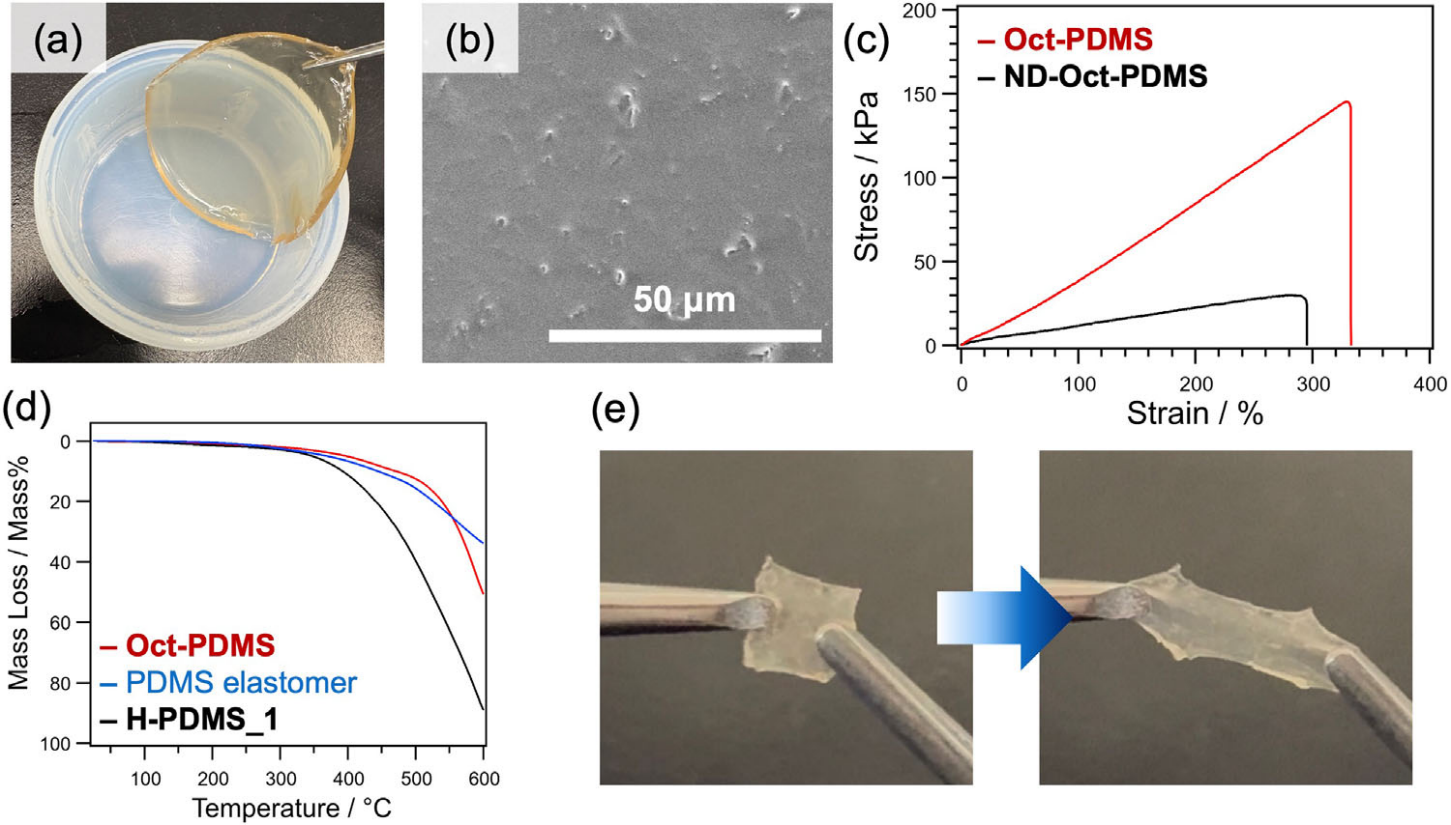
(a) Appearance and (b) cross‐sectional SEM image of **Oct‐PDMS**, (c) Stress‐strain curves of **Oct‐PDMS** and **ND‐Oct‐PDMS**, and (d) TG curves (in N_2_ atmosphere) of **H‐PDMS_1**, **Oct‐PDMS**, and commercially available PDMS elastomer. (e) Stretching of the rejoined cut pieces of TMA silanolate‐incorporated **Oct‐PDMS**.

A suspension of **ViSi_4_‐Oct** in cyclohexane (**ViSi_4_‐Oct_NS**) was used for the nanocomposite formation with PDMS. A transparent elastomer (**Oct‐PDMS**) (Figure 3a) was obtained by Pt‐catalyzed hydrosilylation of **ViSi_4_‐Oct_NS** with SiH‐terminated PDMS (*M*w = 17,200, **H‐PDMS_1**). Unfortunately, the amounts of the SiVi and SiH groups in the mixture of **ViSi_4_‐Oct_NS** and **H‐PDMS_1** were too small to spectroscopically monitor the progress of the reaction. Therefore, a similar reaction was conducted using SiH‐terminated PDMS with a lower molecular weight (*M*w = 4500, **H‐PDMS_2**), and the formation of Si─CH_2_CH_2_─Si linkages was confirmed by solid‐state ^13^C MAS NMR analysis (Supporting Information, Figure S7). It should be noted here that simple mixing of **ViSi_4_‐Oct_NS** and **H‐PDMS_1** without a Pt catalyst resulted in a viscous liquid (data not shown), suggesting that covalent cross‐linking by hydrosilylation is essential for the formation of the elastomer. The SEM image of the cut surface of **Oct‐PDMS** (Figure 3b) showed that **ViSi_4_‐Oct_NS**‐derived particles were dispersed throughout the elastomer, although nanosheets were not clearly visible.

Tensile testing (Figure 3c) confirmed that **Oct‐PDMS** exhibits high flexibility. For comparison, an elastomer was prepared using non‐delaminated octosilicate (**ViMe_2_Si‐Oct**) and **H‐PDMS_1** under otherwise identical conditions. Unlike **Oct‐PDMS**, a translucent elastomer containing some agglomerated particles was obtained (**ND‐Oct‐PDMS**, Supporting Information, Figure S8). The maximum stress of **Oct‐PDMS** was significantly higher than that of **ND‐Oct‐PDMS**, which can be attributed to the increased cross‐linking density resulting from the delamination of the layers.

The thermogravimetric (TG) analysis under N_2_ atmosphere (Figure 3d) revealed that the 5% weight loss temperature of **Oct‐PDMS** was 399°C, which was much higher than that for **H‐PDMS_1** (348°C) and comparable to the commercial PDMS elastomer (370°C) prepared using SYLGARD 184 Silicone Elastomer Kit (Sigma Aldrich) containing silica nanoparticle fillers. This suggests that **ViSi_4_‐Oct** in the elastomer acts as an inorganic filler to impart high thermal stability.

To impart self‐healing ability to **Oct‐PDMS**, silanolate (SiO^−^) groups were incorporated by immersing the elastomer in 0.25 M tetramethylammonium hydroxide (TMAOH) in THF and methanol for 10 min, followed by drying under reduced pressure. In the Fourier transform infrared spectroscopy (FT‐IR) spectrum, small peaks assigned to TMA cations were observed (Supporting Information, Figure S9). This elastomer was then cut in half with a knife, and the cut surfaces were gently pressed together. After heating in an oven at 45°C for 1 day, the cut pieces appeared to rejoin and could be stretched using tweezers (Figure 3e). Zheng and McCarthy reported the self‐healing of partially cross‐linked PDMS elastomers containing terminal silanolate groups through siloxane equilibration.^[^ ^27^ ^]^ The main drawbacks of this self‐healing mechanism are weight loss due to the evaporation of low‐molecular‐weight cyclic dimethylsiloxanes and reduced dimensional stability of the elastomer. We expect that the nanocomposite formation with crystalline silica nanosheets offers a promising approach to address these issues by enhancing the barrier and mechanical properties.

## 3 Conclusion

The interlayer surfaces of layered octosilicate were modified with oligodimethylsiloxane containing a terminal vinyl group. Delamination of the silylated layers was achieved by simple stirring in non‐polar organic solvents. The hydrosilylation reaction of the delaminated nanosheets, modified with vinyl‐terminated oligosiloxanes, with SiH‐terminated PDMS resulted in the formation of a transparent nanocomposite elastomer with enhanced thermal stability. These results demonstrated the dual functionality of the delaminated nanosheets as both cross‐linkers and inorganic nanofillers. Furthermore, self‐healing properties were imparted by immersion of the nanocomposite elastomer in a TMAOH solution. The oligodimethylsiloxane‐grafted silica nanosheets show promise as new 2D nanobuilding blocks for producing a variety of functional siloxane‐based nanomaterials.

## 4 Experimental Section

### Materials

SiO_2_ (fumed silica, S5130) was purchased from Sigma Aldrich. Sodium hydroxide (NaOH, ≥97.0%), toluene (super dehydrated, ≥99.5%), pyridine (dehydrated, ≥99.5%), dichloromethane (≥99.5%), acetonitrile (MeCN, super dehydrated, ≥99.8%), *N,N*‐dimethylformamide (DMF, super dehydrated, ≥99.5%), cyclohexane (≥98.0%), and tetrahydrofuran (THF, with stabilizer, ≥97.0%) were purchased from FUJIFILM Wako Pure Chemical Corporation. Hexadecyltrimethylammonium chloride (C_16_TMACl, >95.0%), hexamethylcyclotrisiloxane (D_3_, >98.0%), dimethyl‐*n*‐octylchlorosilane (C_8_Me_2_SiCl, >96.0%), dimethylvinylchlorosilane (ViMe_2_SiCl, >97.0%), platinum(0)‐1,3‐divinyltetramethyldisiloxane complex (Karstedt's catalyst, 19.0%–21.5% as Pt), and tetramethylammonium hydroxide (TMAOH, 10% in methanol) were purchased from Tokyo Chemical Industry Co., Ltd. Hydride terminated PDMS (**H‐PDMS_1**, molecular weight: 17,200, viscosity: 500 cSt) and monodisperse hydride terminated PDMS (**H‐PDMS_2**, molecular weight: 4500, viscosity: 50 cSt) were purchased from Gelest Inc. All these chemicals were used without further purification.

### Preparation of C_16_TMA‐exchanged layered octosilicate (C_16_TMA‐Oct)

Layered octosilicate (Na_8_Si_32_O_64_(OH)_8_·32H_2_O, Na‐Oct) and **C_16_TMA‐Oct** were synthesized according to a previous report.^[^ ^14^ ^]^ Fumed silica (SiO_2_), NaOH, and pure water were mixed with a molar ratio of 4:1:25.8 and stirred for 1 h, followed by 1 h of aging. Na‐Oct seed crystals were added to the mixture, and hydrothermal treatment was performed for 4 weeks. The resulting product was washed three times with pure water, yielding a white powder. Na‐Oct was stirred in an aqueous solution of C_16_TMACl (0.1 M) at room temperature for 1 day and was collected by centrifugation. This procedure was repeated three times so that the interlayer Na^+^ could be fully exchanged with C_16_TMA cations. Finally, the product was washed with pure water three times to obtain a white powder (**C_16_TMA‐Oct**).

### Synthesis of vinyl‐ and SiCl‐terminated linear tetra(dimethylsiloxane) (ViSi_4_Cl)

Vinyl‐ and SiCl‐terminated linear tetra(dimethylsiloxane) (CH_2_═CHSiMe_2_OSiMe_2_OSiMe_2_OSiMe_2_Cl, **ViSi_4_Cl**) was synthesized according to the literature method.^[^ ^28^ ^]^ In a Schlenk flask, D_3_ (18 g, 0.081 mol), ViSiMe_2_Cl (10.97 mL, 0.081 mol), anhydrous MeCN (7.8 mL), and anhydrous DMF (0.6 mL) were mixed and stirred at 25°C for 3 h in a nitrogen atmosphere. After removal of the solvents under vacuum, vacuum distillation at 90°C gave **ViSi_4_Cl** as a colorless, clear liquid. ^1^H NMR (500.16 MHz, chloroform‐*d*): *δ* (ppm) = 0.07 (s, 6H; OSi(C*H*_3_)_2_OSi(CH_3_)_2_Cl), 0.13 (s, 6H; H_2_C═CHSi(CH_3_)_2_OSi(C*H*_3_)_2_), 0.16 (s, 6H; H_2_C═CHSi(C*H*_3_)_2_), 0.45 (s, 6H; OSi(C*H*_3_)_2_Cl), 5.71–5.76 (dd, 1H; *H^cis^* C(H)═CHSi), 5.92–5.96 (dd, 1H; *H* ^*trans*^C(H)═CHSi), 6.09–6.16 (dd, 1H; H_2_C═C*H*Si); ^13^C NMR (125.77 MHz, chloroform‐*d*): *δ* (ppm) = 0.27 (OSi(*C*H_3_)_2_OSi(CH_3_)_2_Cl), 0.94 (H_2_C═CHSi(CH_3_)_2_OSi(*C*H_3_)_2_), 1.13 (H_2_C═CHSi(*C*H_3_)_2_OSi), 4.09 (SiOSi(*C*H_3_)_2_Cl), 131.74 (H_2_C═*C*HSi(CH_3_)_2_OSi), 139.27 (H_2_ *C*═CHSi(CH_3_)_2_OSi); ^29^Si NMR (99.37 MHz): *δ* (ppm) = −20.33 (H_2_C═CHSi(CH_3_)_2_O*Si*(CH_3_)_2_), −19.13 (O*Si*(CH_3_)_2_OSi(CH_3_)_2_Cl), −3.83 (H_2_C═CH*Si*(CH_3_)_2_O), 3.63 (O*Si*(CH_3_)_2_Cl). The ^1^H, ^13^C, and ^29^Si NMR spectra are shown in the Supporting Information (Figure S10).

### Preparation of layered octosilicate modified with vinyl‐terminated tetra(dimethylsiloxane)

In a Schlenk flask, **C_16_TMA‐Oct** (1.00 g) was dried under vacuum at 120°C for 3 h. **ViSi_4_Cl** (6.31 g, 0.018 mol), anhydrous toluene (25 mL), and anhydrous pyridine (1.5 mL, 0.019 mol) were added to the flask under a nitrogen atmosphere. The molar ratio was adjusted to SiOH/O^−^:**ViSi_4_Cl**:pyridine = 1:5:5. Silylation reaction was performed by stirring the mixture at room temperature for 1 day. The resulting mixture was centrifuged, and the supernatant was removed. C_16_TMACl (byproduct), unreacted oligomeric siloxanes, pyridine hydrochloride, and other impurities were removed by repeated washing with dichloromethane (three times) and hexane (three times). The solids were collected by centrifugation (5000 rpm, 5 min) and dried under reduced pressure, yielding a white powder (**ViSi_4_‐Oct**).

### Delamination of ViSi_4_‐Oct in organic solvents

**ViSi_4_‐Oct** (0.60 g) was added to cyclohexane (30 mL), and the mixture was stirred using a magnetic stirrer (600 rpm) at room temperature for 2 days, resulting in a translucent suspension (**ViSi_4_‐Oct_NS**). Similarly, **ViSi_4_‐Oct** (0.01 g) was added to *n*‐hexane (1 mL) and toluene (1 mL), and the mixtures were stirred (600 rpm) at room temperature for 3 days.

### Preparation of a nanocomposite elastomer by hydrosilylation of ViSi_4_‐Oct_NS with H‐PDMS_1

**H‐PDMS_1** (2.77 g) was dried under vacuum for 30 min, and then it was mixed with **ViSi_4_‐Oct_NS** (5.87 mL) at a molar ratio of SiVi:SiH = 2:5 under a nitrogen atmosphere. A Karstedt's catalyst (0.53 µL) was added, and the mixture was stirred at room temperature for 1 h. Then the mixture was transferred to a cylindrical perfluoroalkoxy alkane container in a separable flask under a nitrogen atmosphere and heated at 60°C for 2 days. Finally, the solvent was removed under vacuum, and a pale‐yellow transparent elastomer (**Oct‐PDMS**) was obtained. The reaction of **ViSi_4_‐Oct_NS** with **H‐PDMS_2** was also conducted using the same procedure. The resulting solid–liquid mixture was washed with hexane to obtain a white powder.

### Characterization

Liquid‐state ^1^H, ^13^C, and ^29^Si NMR spectra were recorded on a JEOL JNM ECZ500 spectrometer with resonance frequencies of 500.16 MHz, 125.77 MHz, and 99.37 MHz, respectively, using a 5 mmφ glass tube. Chloroform‐*d* was used to obtain lock signals. Tetramethylsilane (*δ* = 0 ppm) was used as the internal reference. For ^29^Si NMR, a small amount of Cr(acac)_3_ (acac = acetylacetonate) was added as a relaxation agent. Powder XRD patterns were obtained by a RIGAKU Rint‐Ultima III diffractometer using Cu K*α* radiation (40 kV, 40 mA). XRD patterns for investigation of swelling behavior were obtained by a RIGAKU Rint‐Ultima IV diffractometer using Fe K*α* radiation (40 kV, 30 mA). Solid‐state ^13^C CP/MAS NMR spectra were recorded on a JEOL JNM ECA400 spectrometer at a resonance frequency of 100.53 MHz with a pulse delay of 5 s and a contact time of 5 ms. Solid‐state ^13^C MAS NMR spectrum was recorded on the same spectrometer with a 90° pulse and a pulse delay of 15 s. Solid‐state ^29^Si MAS NMR spectra were also recorded on the same spectrometer at a resonance frequency of 79.43 MHz with a 90° pulse and a recycle delay of 500 s.^[^ ^29^ ^]^ The samples for solid‐state NMR were put in a 5 mmφ zirconia rotor and spun at 8 kHz. For solid‐state ^13^C NMR and ^29^Si NMR, hexamethylbenzene (*δ* = 17.4 ppm) and poly(dimethylsilane) (*δ* = −33.8 ppm) were used as external references, respectively. FT‐IR spectroscopy was performed using a JASCO FT/IR‐6100 spectrometer. The FT‐IR spectra of liquid and elastic samples were obtained using the attenuated total reflection (ATR) method with an ATR accessory (JASCO ATR PRO ONE) with a diamond prism. SEM images were obtained on a Hitachi S‐5500 electron microscope with an accelerating voltage of 1 kV. For SEM observation of powder samples, a small amount of the powder was adhered to a carbon tape. An elastomer sample was cut with a knife, and the cut surface was observed. TEM images were obtained on a JEOL JEM‐2010 microscope with an accelerating voltage of 200 kV. For SEM and TEM observation of delaminated samples, the suspension was drop‐cast and dried on carbon‐coated Cu microgrids. AFM images were obtained on an Oxford Instruments Jupiter‐XR by the tapping mode using a Si probe (AC160TS). The suspensions of delaminated samples were diluted 100‐fold with hexane, followed by drop‐casting on a Si substrate and drying under reduced pressure for the AFM observation. A tensile test of elastomer samples was performed using SHIMAZU EZ‐SX 200N. Dumbbell pieces for the tensile tests were prepared using an SD Lever Type Sample Cutting Machine SDL‐100 equipped with a Super Dumbbell cutter SDMP‐1000‐D (Dumbbell Co., Ltd., JIS K‐6251‐7). TG analyses were conducted using a Rigaku Thermo plus EVO2 TG8121 under nitrogen flow at a heating rate of 10°C min^−1^. The partial structural models were displayed using the VESTA software.^[^ ^30^ ^]^

## Supporting Information

Additional experimental details, and characterization data including liquid‐state and solid‐state NMR spectra, FT‐IR spectra, XRD patterns, AFM images, and SEM images are available in the Supporting Information.

## Conflicts of Interest

The authors declare no conflict of interest.

## Supporting information



Supporting Information

## Data Availability

The data that support the findings of this study are available from the corresponding author upon reasonable request.
