# Supplementary material for: Interlayer Modification of Crystalline Layered Silicates with Oligodimethylsiloxane
Source: Chemistry. 2025 Apr 13;31(25):e202500262. doi: 10.1002/chem.202500262 (PMC12057598; doi:10.1002/chem.202500262)
Supplement: Supplementary file 1 — Supporting Information [file CHEM-31-e202500262-s001.docx]

**Supporting Information**

**Interlayer Modification of Crystalline Layered Silicates**

**with Oligodimethylsiloxane**

Riho Wakino, Mai Suzuki, Yoshiaki Miyamoto, Masashi Yatomi, Takamichi Matsuno, and Atsushi Shimojima*

Department of Applied Chemistry, Faculty of Science and Engineering, Waseda University, 3-4-1 Okubo, Shinjuku-ku, Tokyo 169-8555, Japan.

Kagami Memorial Research Institute for Materials Science and Technology, Waseda University, 2-8-26 Nishiwaseda, Shinjuku-ku, Tokyo 169-0051, Japan.

Waseda Research Institute for Science and Engineering, Waseda University, 3-4-1 Okubo, Shinjuku-ku, Tokyo 169-8555, Japan

Email: shimojima@waseda.jp

Table of Contents

[1. Experimental Details 3](#_Toc194261889)

[**Procedure S1.** Silylation of **C_16_TMA-Oct** with dimethylvinylchlorosilane (ViMe_2_SiCl) and dimethyl-*n*-octylchlorosilane (C_8_Me_2_SiCl) 3](#_Toc194261890)

[2. Supporting data 3](#_Toc194261891)

[**Figure S1** Additional SEM images of **ViSi_4_-Oct** 3](#_Toc194261892)

[**Figure S2** Typical AFM images and the corresponding height profiles for **ViSi_4_-Oct** after stirring in cyclohexane for 2 days 4](#_Toc194261893)

[**Figure S3** (a) XRD patterns of **ViSi_4_-Oct** before and after dropping *n*-hexane and toluene. In the case of *n*-hexane, a peak at 2.1 nm was still observed, which was possibly due to the evaporation of *n*-hexane during the measurement. (b) Photograph and (c) SEM image of **ViSi_4_-Oct** dispersed in *n*-hexane. (d) Photograph and (e) SEM image of **ViSi_4_-Oct** dispersed in toluene. 4](#_Toc194261894)

[**Figure S4** Solid-state ^29^Si MAS NMR spectra of (a) **ViMe_2_Si-Oct** and (b) **C_8_Me_2_Si-Oct**. 5](#_Toc194261895)

[**Figure S5** (a) XRD patterns of **ViMe_2_Si-Oct** and **C_8_Me_2_Si-Oct** before (black) and after (red) dropping cyclohexane on the powders. (b) Appearance of the dispersion (after standing for 1 day) and (c) SEM image of **C_8_Me_2_Si-Oct** after stirring in cyclohexane for 3 days. (d) Appearance of the dispersion (after standing for 1 day) and (e) SEM image of **ViMe_2_Si-Oct** after stirring in cyclohexane for 3 days. 5](#_Toc194261896)

[**Figure S6** Typical AFM images and the corresponding height profiles for **C_8_Me_2_Si-Oct** after stirring in cyclohexane for 3 days. 6](#_Toc194261897)

[**Figure S7** ^13^C MAS NMR spectra of (a) **ViSi_4_-Oct** and (b) **Oct-PDMS** (*M*n = 4500). 6](#_Toc194261898)

[**Figure S8** Appearance of the elastomer (**ND-Oct-PDMS**), prepared by the hydrosilylation reaction between 7](#_Toc194261899)

[**Figure S9** FT-IR spectra of (a) **Oct-PDMS** before and after the treatment with TMAOH. 7](#_Toc194261900)

[**Figure S10** (a)^1^H NMR, (b) ^13^C NMR, and (c) ^29^Si NMR spectra of **ViSi_4_Cl** (in CDCl_3_). 7](#_Toc194261901)

# 1. Experimental Details

## **Procedure S1.** Silylation of **C_16_TMA-Oct** with dimethylvinylchlorosilane (ViMe_2_SiCl) and dimethyl-*n*-octylchlorosilane (C_8_Me_2_SiCl)

In a Schlenk flask, **C_16_TMA-Oct** (0.60 g) was dried under vacuum at 120 °C for 3 h. ViMe_2_SiCl (1.50 mL, 0.011 mol), anhydrous toluene (30 mL), and anhydrous pyridine (7.5 mL) were added to the flask under a nitrogen atmosphere. The molar ratio was adjusted to SiOH/O^−^ : ViMe_2_SiCl = 1 : 5. Silylation reaction was performed by stirring the mixture at room temperature for 1 day. The resulting mixture was centrifuged, and the supernatant was removed. C_16_TMACl (byproduct), oligomeric siloxanes, pyridine hydrochloride, and other impurities were removed by repeated washing with dichloromethane (three times) and hexane (three times). The solids were collected by centrifugation (5,000 rpm, 5 min) and dried under reduced pressure, yielding a white powder (**ViMe_2_Si-Oct**).

Silylation of **C_16_TMA-Oct** with C_8_Me_2_SiCl was performed by adding C_8_Me_2_SiCl (3.87 mL, 0.016 mol), anhydrous toluene (30 mL), and anhydrous pyridine (10 mL) to dried **C_16_TMA-Oct** (0.50 g) under a nitrogen atmosphere. The molar ratio was adjusted to SiOH/O^−^ : C_8_Me_2_SiCl = 1 : 10. Other procedures are the same as those for preparing **ViMe_2_Si-Oct**, yielding a white powder (**C_8_Me_2_Si-Oct**).

# 2. Supporting data


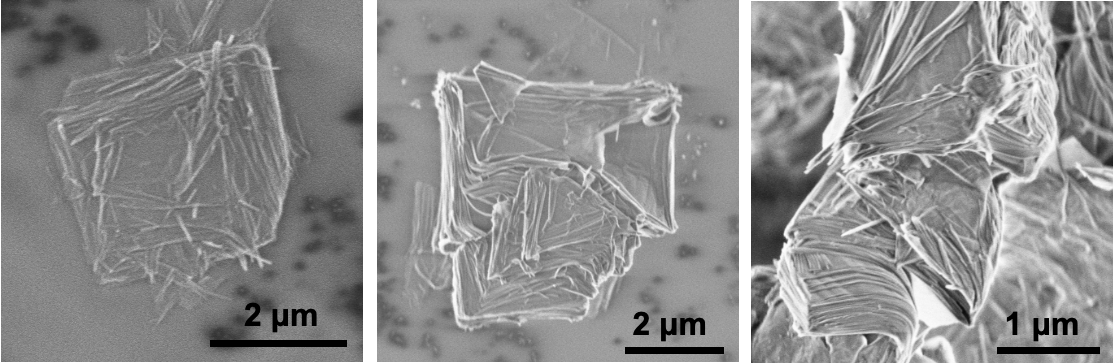


**Figure S1** Additional SEM images of **ViSi_4_-Oct**.


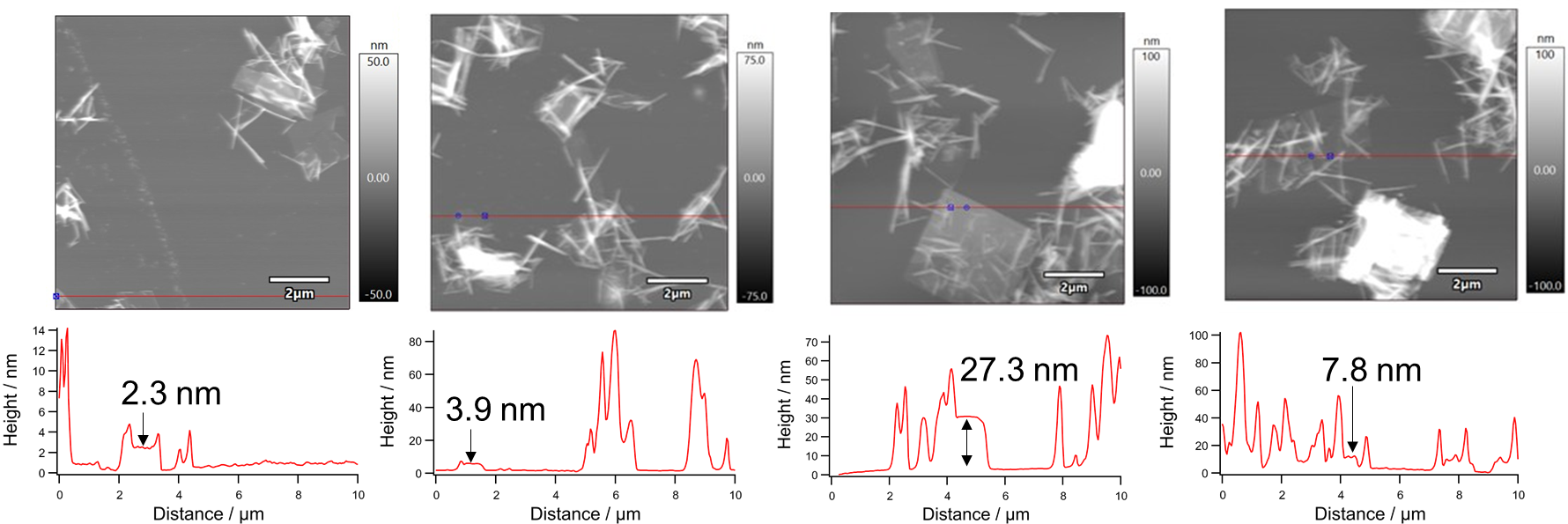


**Figure S2** Typical AFM images and the corresponding height profiles for **ViSi_4_-Oct** after stirring in cyclohexane for 2 days (**ViSi_4_-Oct_NS**). The suspension was diluted tenfold with hexane and then cast on Si substrates for the measurements.


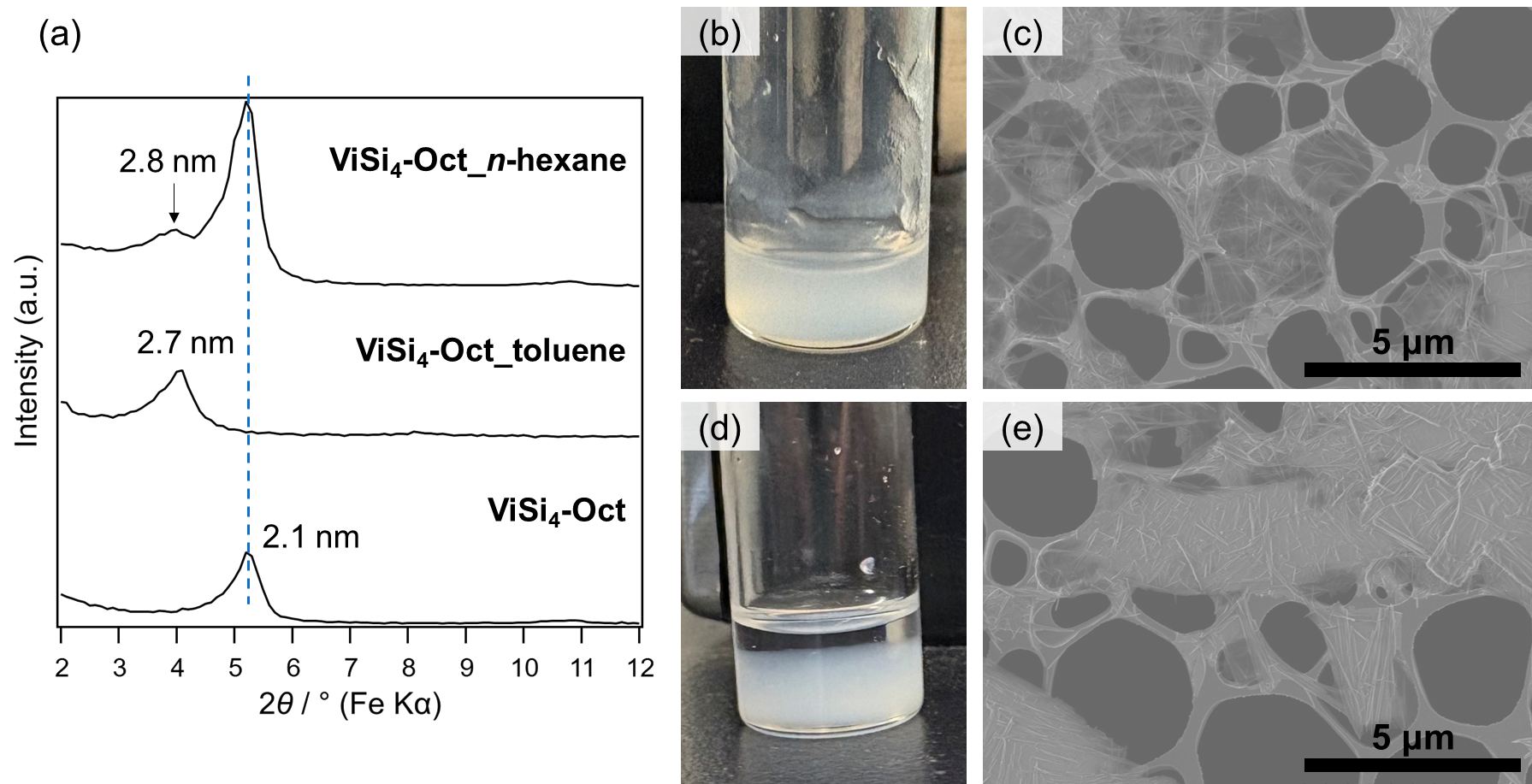


## **Figure S3** (a) XRD patterns of **ViSi_4_-Oct** before and after dropping *n*-hexane and toluene. In the case of *n*-hexane, a peak at 2.1 nm was still observed, which was possibly due to the evaporation of *n*-hexane during the measurement. (b) Photograph and (c) SEM image of **ViSi_4_-Oct** dispersed in *n*-hexane. (d) Photograph and (e) SEM image of **ViSi_4_-Oct** dispersed in toluene.


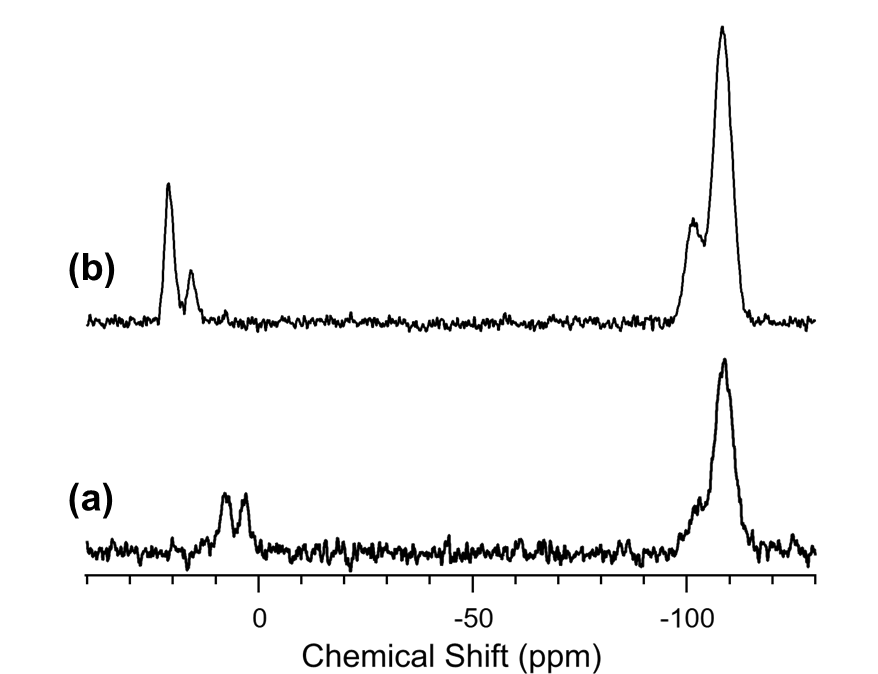


**Figure S4** Solid-state ^29^Si MAS NMR spectra of (a) **ViMe_2_Si-Oct** and (b) **C_8_Me_2_Si-Oct**. The signals observed at 3 ppm and 8 ppm for **ViMe_2_Si-Oct**, and those observed at 16 ppm and 21 ppm for **C_8_Me_2_Si-Oct**, correspond to the silyl groups immobilized on the layer surface.


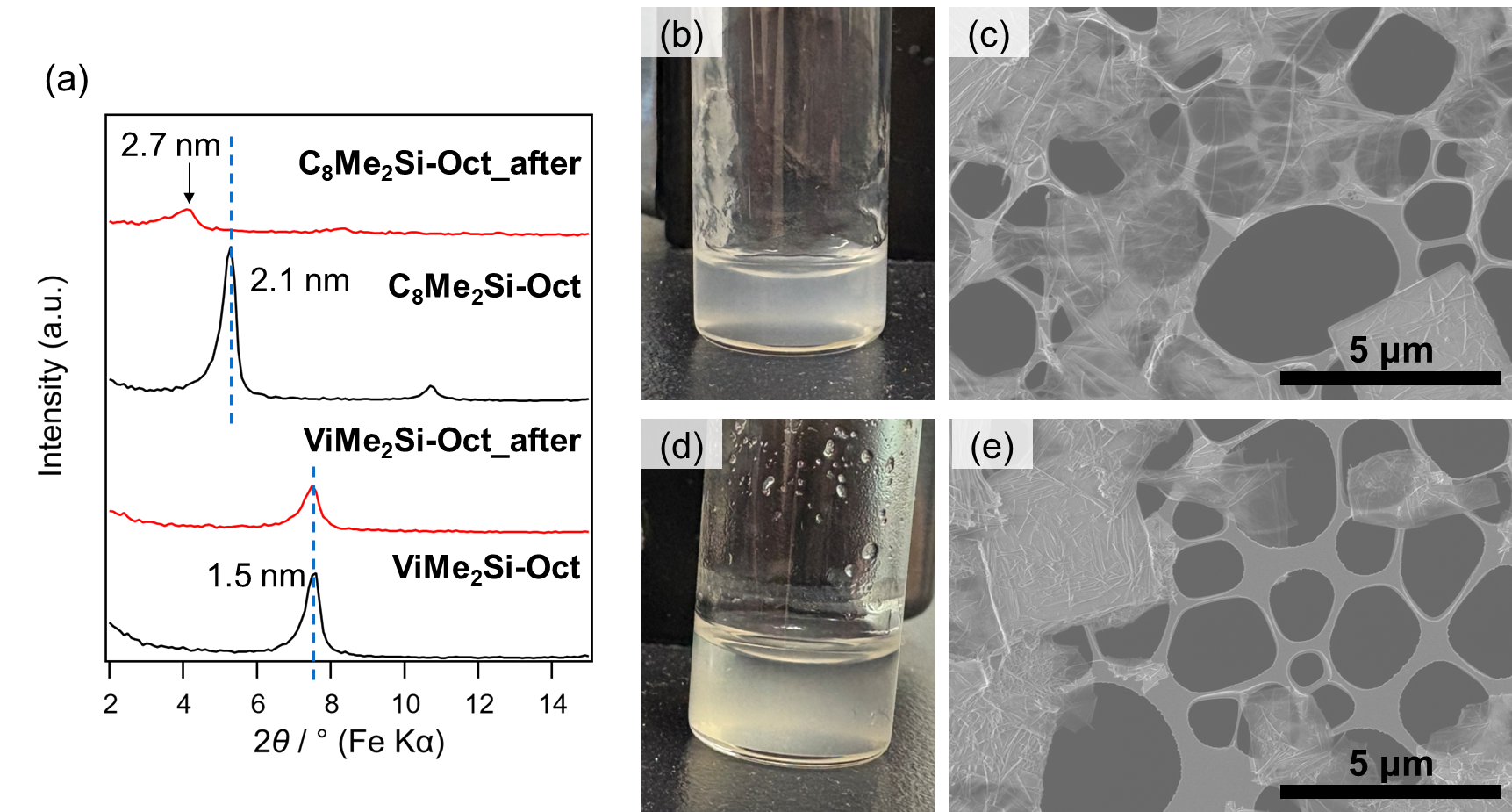


## **Figure S5** (a) XRD patterns of **ViMe_2_Si-Oct** and **C_8_Me_2_Si-Oct** before (black) and after (red) dropping cyclohexane on the powders. (b) Appearance of the dispersion (after standing for 1 day) and (c) SEM image of **C_8_Me_2_Si-Oct** after stirring in cyclohexane for 3 days. (d) Appearance of the dispersion (after standing for 1 day) and (e) SEM image of **ViMe_2_Si-Oct** after stirring in cyclohexane for 3 days.


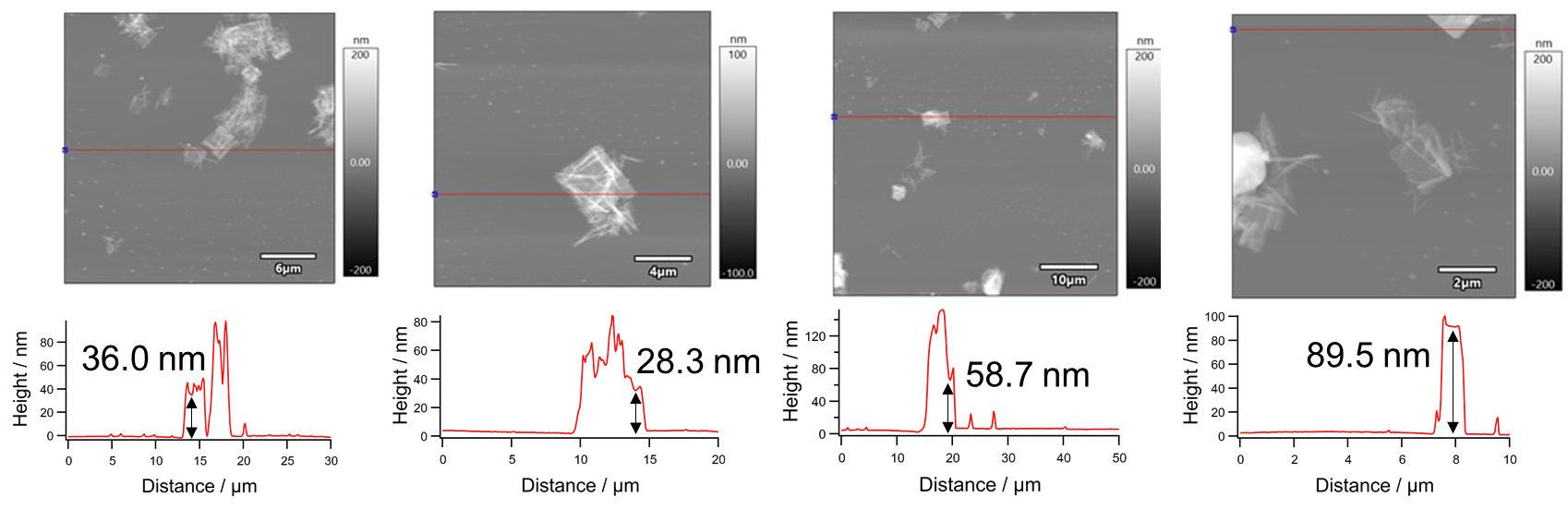


**Figure S6** Typical AFM images and the corresponding height profiles for **C_8_Me_2_Si-Oct** after stirring in cyclohexane for 3 days. The suspension was diluted 100-fold with hexane and cast on Si substrates for the measurements.


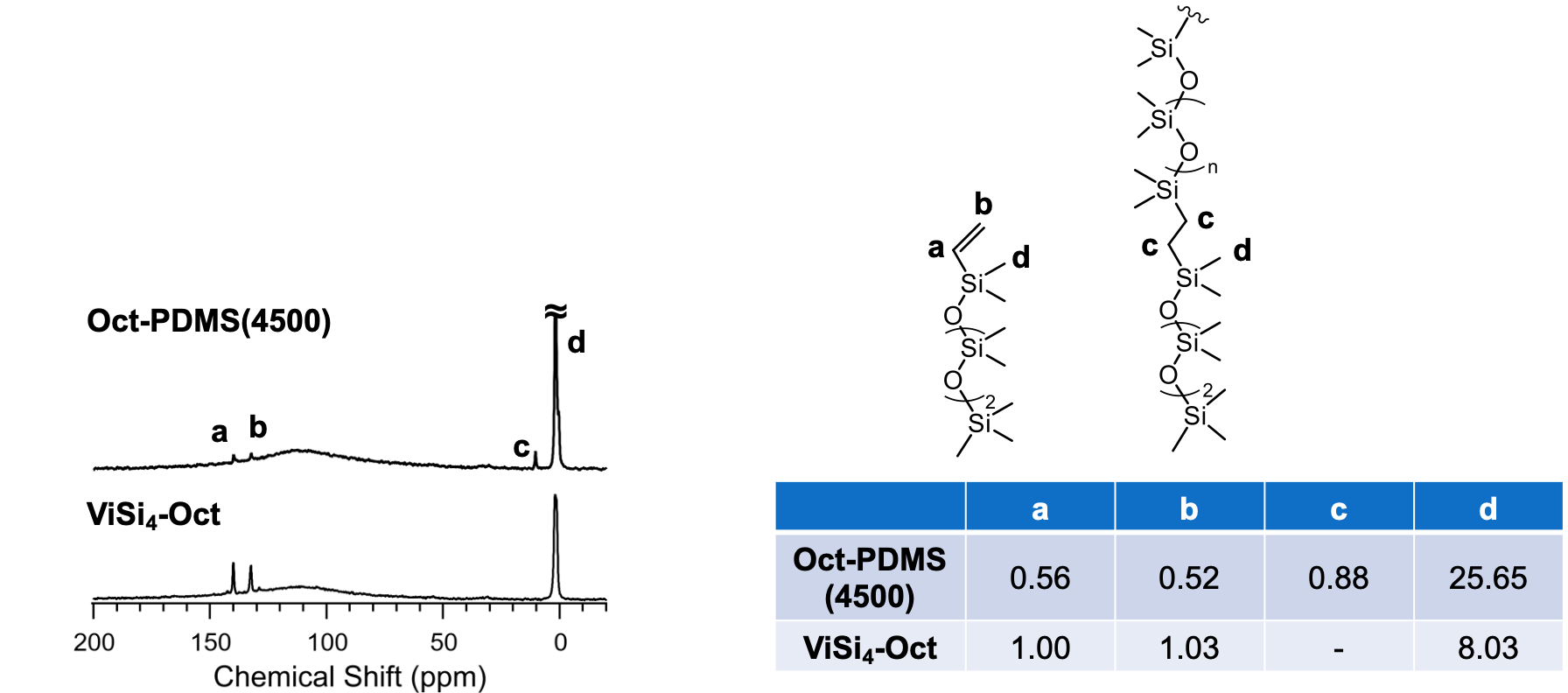


**Figure S7** ^13^C MAS NMR spectra of (a) **ViSi_4_-Oct** and (b) **Oct-PDMS** (*M*n = 4500). To clearly observe the bands of the terminal vinyl groups, SiH-terminated PDMS with lower molecular weight (*M*n = 4500) was used for the hydrosilylation reaction. After the hydrosilylation reaction, unreacted PDMS were removed by washing with hexane.


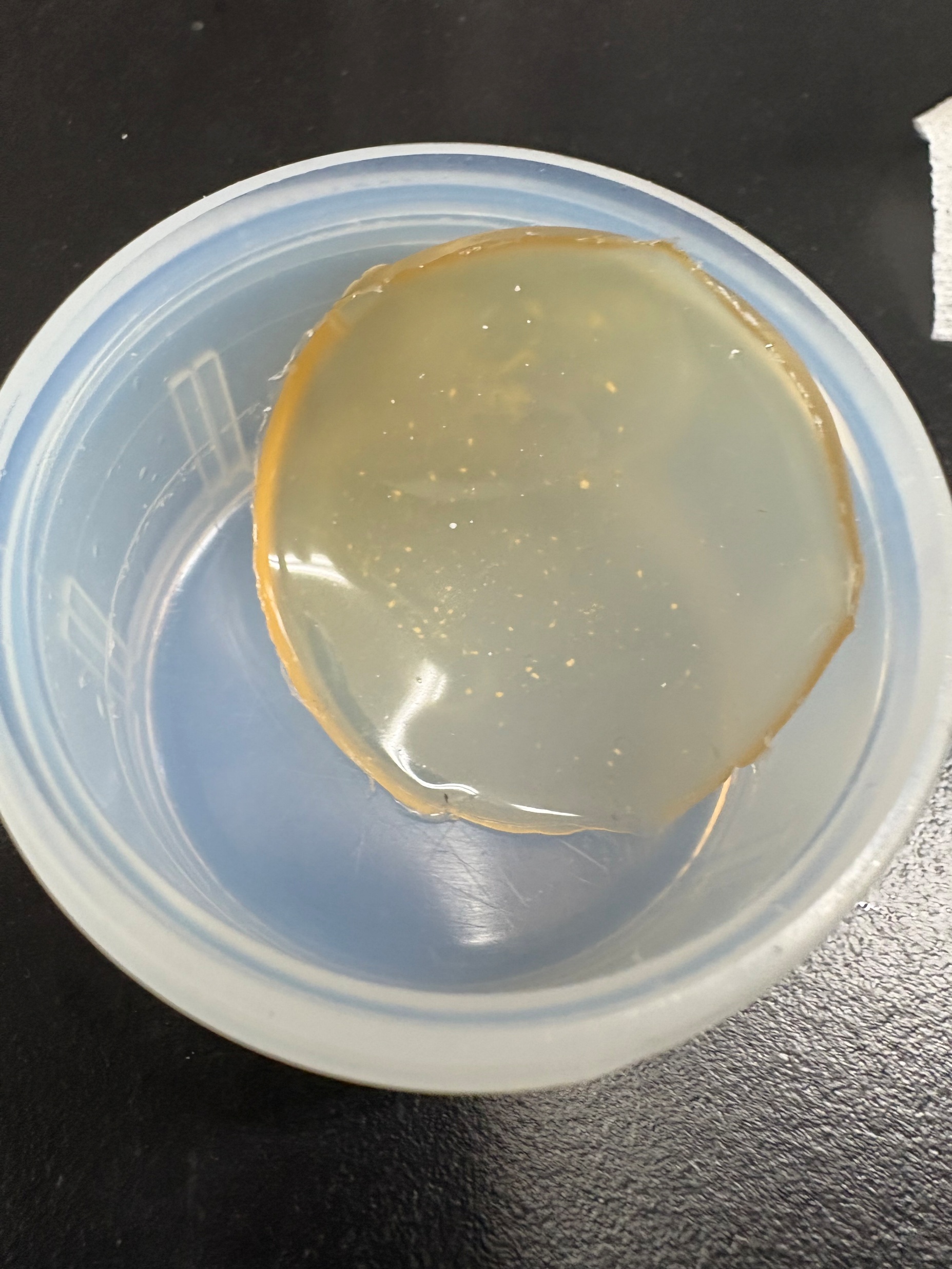


**Figure S8** Appearance of the elastomer (**ND-Oct-PDMS**), prepared by the hydrosilylation reaction between **ViMe_2_Si-Oct** and **H-PDMS_1**. The elastomer is less transparent than **Oct-PDMS** (cf. Figure 3(a)), and agglomerated white particles are observed.


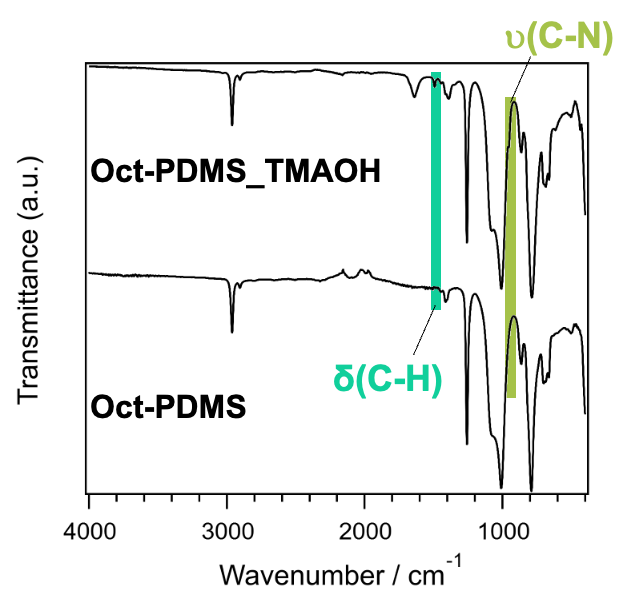


## **Figure S9** FT-IR spectra of (a) **Oct-PDMS** before and after the treatment with TMAOH.


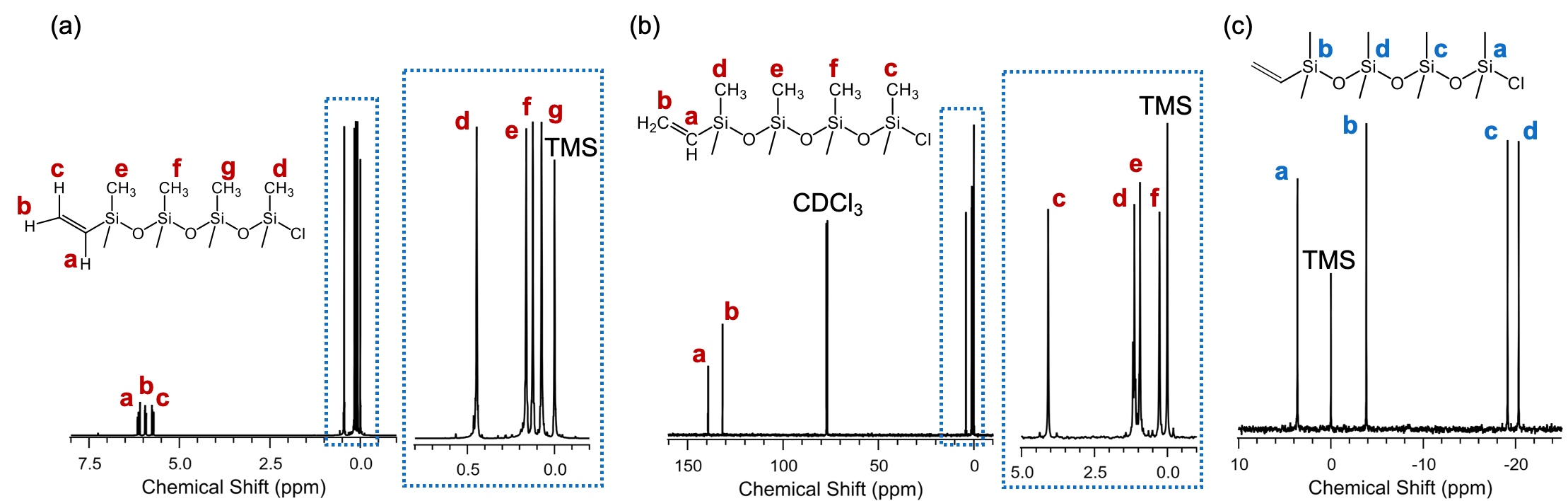


## **Figure S10** (a)^1^H NMR, (b) ^13^C NMR, and (c) ^29^Si NMR spectra of **ViSi_4_Cl** (in CDCl_3_).
